# Application of Fungal Biomass for the Development of New Polylactic Acid-Based Biocomposites

**DOI:** 10.3390/polym14091738

**Published:** 2022-04-24

**Authors:** Mohammadtaghi Asadollahzadeh, Amir Mahboubi, Mohammad J. Taherzadeh, Dan Åkesson, Patrik R. Lennartsson

**Affiliations:** Swedish Centre for Resource Recovery, University of Borås, 501 90 Borås, Sweden; amir.mahboubi_soufiani@hb.se (A.M.); mohammad.taherzadeh@hb.se (M.J.T.); dan.akesson@hb.se (D.Å.); patrik.lennartsson@hb.se (P.R.L.)

**Keywords:** fungal biomass (FB), poly(lactic acid) (PLA), triethyl citrate (TEC), biopolymers, biocomposite, brittleness

## Abstract

Fungal biomass (FB), a by-product of the fermentation processes produced in large volumes, is a promising biomaterial that can be incorporated into poly(lactic acid) (PLA) to develop enhanced biocomposites that fully comply with the biobased circular economy concept. The PLA/FB composites, with the addition of triethyl citrate (TEC) as a biobased plasticizer, were fabricated by a microcompounder at 150 °C followed by injection molding. The effects of FB (10 and 20 wt %) and TEC (5, 10, and 15 wt %) contents on the mechanical, thermal and surface properties of the biocomposites were analyzed by several techniques. The PLA/FB/TEC composites showed a rough surface in their fracture section. A progressive decrease in tensile strength and Young’s modulus was observed with increasing FB and TEC, while elongation at break and impact strength started to increase. The neat PLA and biocomposite containing 10% FB and 15% TEC exhibited the lowest (3.84%) and highest (224%) elongation at break, respectively. For all blends containing FB, the glass transition, crystallization and melting temperatures were shifted toward lower values compared to the neat PLA. The incorporation of FB to PLA thus offers the possibility to overcome one of the main drawbacks of PLA, which is brittleness.

## 1. Introduction

In spite of the fact that the use of petroleum-based polymers for the production of composites is convenient in terms of fulfilling the performance requirements of many applications, it faces three serious challenges including depleting fossil resources, increasing oil prices and increasing environmental concerns for their disposal. Therefore, the use of polymers derived from renewable resources (biobased polymers) is one of the potential solutions in combating these challenges as they can be effortlessly recycled or disposed of after their service life without harming the environment [1,2,3]. A lot of studies have been performed to replace the commodity petro-based polymers with biobased polymers such as poly(lactic acid) (PLA) [4], polyhydroxyalkanoates (PHAs) [5], polybutylene succinate (PBS) [6], polycaprolactone (PCL) [7], starch [8] and their composites [9] in different applications.

Among the biopolymers, PLA is one of the most promising commercially available polymers on the market and widely used in many areas of science, engineering and technology, ranging from biomedicine to packaging [10,11,12]. The global PLA market extent was priced at USD 0.5 billion in 2020 and is set to increase with an expected consecutive annual growth rate (CAGR) of 18.1% from 2021 to 2028 [10]. PLA is comparable to those of traditional petroleum-based polymers in terms of strength, stiffness and optical transparency. Nevertheless, the application of PLA is still restricted because of its inherent brittleness, low thermal stability, slow crystallization rate, poor impact strength, and relatively high production cost [2,11,13,14]. Until now, a variety of strategies including the use of different types of reinforcements and grafting, plasticization, or blending of PLA with other polymers have been developed in the literature to overcome these drawbacks [10,14,15].

Biocomposites can be prepared by compounding biopolymers with natural fibers or fillers, which may combine the advantages of both components and may have better properties than either component [16,17]. Blending PLA with inexpensive natural polymers such as starch [9,18,19,20], soy protein (SP) [21,22,23], agricultural and food wastes [4,24,25,26,27], wood fiber/flour [28,29,30], waste (recycled) paper [31,32,33,34,35], chicken feather [36,37,38,39,40] and leather waste [15] could also substantially lower the total cost while improving some of the properties of PLA.

Fungal biomass (FB) as a major by-product stream from the fermentation processes is formed of a branched fibrous structure which is called mycelium. Fungal mycelium typically consists of microscopic filaments (hyphae) with a diameter of 1–30 μm, depending on the species and growth environment, and lengths of a few microns to several meters [41]. FB is generally comprised of protein, lipids, polysaccharides, chitin (and/or chitosan) and inorganic salts in different portions [42]. The large quantities of functional groups present in FB, such as hydroxyl, amine, and carboxyl groups, make it a promising candidate for conversion to new operational biobased materials as these functional groups enable chemical interactions with other organic fibers, fillers or additives and thus improve the required properties of the final biobased materials [43]. FB can be produced via the fermentation of most organic wastes and residuals, and therefore, it has a great potential for large-scale production. For example, the worldwide fermentation of citric acid using filamentous fungous *Aspergillus niger* results in the generation of 80,000 tons/year of the FB as by-product [44]. Moreover, unlike biopolymers of plant origin such as soy protein and corn starch, FB is available all around the year, as its production is neither seasonal nor climate (geographical location)-dependent [45].

Earlier studies have indicated that fungal mycelium biomass can be used to produce replacements for products such as animal-based leather and its substitutes [46,47], synthetic foams for packaging [48], fibers for textiles [49], and high-performance paper or film-like materials [43,50,51,52,53,54,55,56,57,58] from a range of different fungal biomass sources and fermentation pathways. Although fungal mycelium biomass has been combined with nanocellulose [59,60], plant cellulose [43,50] and pectin [61] to obtain a new blended biomaterial, there are no studies on blended biopolymers from polylactic acid and fungal biomass. In this study, mycelium biomass from filamentous fungous *Aspergillus oryzae* cultivated in submerged fermentation on oat flour was compounded with the PLA in varying amounts to obtain a novel eco-friendly biocomposite.

Previous studies demonstrated that plasticizers must be included when natural polymers such as soy protein and starch are blended with PLA in order to lower the melting temperature of PLA and improve the processability, flexibility and ductility of composites from bio-derived protein and polysaccharides [62,63]. To date, a big number of plasticizers have been investigated for the modification of PLA and its blend. Among them, polyethylene glycol (PEG), glycerol and citrate esters are perhaps the most commonly investigated plasticizers [63]. Triethyl citrate (TEC) as a biodegradable, nontoxic, and efficient plasticizer was used in this work. The influence of TEC on the mechanical, physical and thermal properties of PLA and PLA-based biocomposites has been well studied. Paul et al. (2021) used TEC to improve the dispersion of the microcrystalline cellulose (MCC) in PLA. The developed biocomposites containing TEC showed improved ductility and crystallinity [64]. The modification of PLA by plasticizing agents such as ethoxylated lauryl alcohol, block copolymer of ethylene oxide and propylene oxide, and ethoxylated stearic acid, di-2-ethylhexyl adipate, di-2-ethylhexyl sebacate and TEC has been carried out to improve its plastic deformability [65]. The addition of 10 wt % of TEC to PLA blended with date palm waste resulted in further improvement in the elongation at break of the optimal composite as compared with polybutylene adipate terephthalate (PBAT) [4].

Fungal mycelium biomass produced from organic waste/residues can be a great candidate for the fabrication of biobased materials owing to its complex content mainly built up of fibers, proteins, etc. The unique characteristic of FB signals potential for its application as a blending component in sought-after biocomposites such as that of PLA-based. However, to the knowledge of the authors, the production and characterization of FB-PLA biocomposites have not been investigated to the date. Therefore, in this study, the effects of the addition of FB (10 and 20 wt %) and TEC (5, 10, and 15 wt %) to the PLA matrix on the mechanical, thermal and surface properties of the resulting biocomposites were profoundly investigated.

## 2. Materials and Methods

### 2.1. Materials

**Figure 1:**
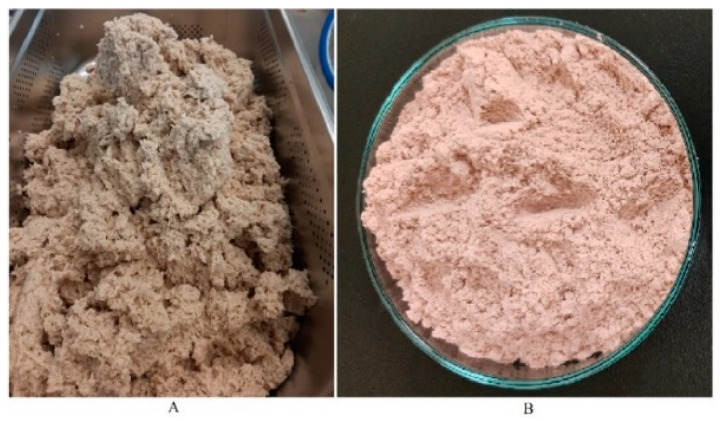
Fungal biomass (FB) (**A**) after washing/dewatering and (**B**) after drying/grinding.

The PLA used for the research was a commercial injection molding grade of PLA IngeoTM 3051D from NatureWorks (Minnetonka, MN, USA). According to the manufacturer’s data, it has a density of 1.24 g/cm^3^, while its melt flow rate (MFR) is 10–30 g/10 min at 190 °C and 2.16 kg load. FB was obtained directly after the submerged fermentation of *Aspergillus oryzae* CBS 819.72 (Centraalbureau voor Schimmelcultures, Utrecht, The Netherlands) on 30 g/L oat flour slurry in a 1200 L airlift bioreactor (Knislinge Mekaniska Verkstad AB, Kristianstad, Sweden). The harvested FB was washed with tap water, dried in a fan-assisted oven at 70 °C, ground using an electric grinder (M 20 Universal mill, IKA, Staufen, Germany) and sieved to 250-micron particles using an electric shaker with the corresponding sieve mesh (HAVER EML 200 Pure, HAVER & BOECKER Co., Oelde, Germany). The composition and appearance of the FB used is presented in Table 1 and Figure 1, respectively. Moisture, ash, total lipid and crude protein contents were measured by the division of Food and Nutrition Science at Chalmers University, Sweden. The plasticizer triethyl citrate (TEC) was purchased from Alfa Aesar (Thermo Fisher GmbH, Kandel, Germany). It is a colorless and odorless oily liquid having a density of 1.136 g/cm^3^, viscosity of 35.2 mPa.s (25 °C) and a molecular weight of 276.29 g/mol.

### 2.2. Preparation of Composites

Before processing, PLA pellets and FB were dried under vacuum at 60 °C overnight. The PLA-based specimens were prepared using a Micro 15cc Twin Screw Compounder (DSM Xplore, Sittard, The Netherlands) paired with a Micro 12cc Injection Molding Machine (DSM Xplore, Sittard, The Netherlands). All composite specimens were processed at 150 °C with a screw speed of 100 rpm while neat PLA processing was completed at 180 °C with the same speed. Argon gas was passed into the barrel of the microcompounder during the processing in order to prevent the degradation of the polymers. The composites were fabricated in such a way that PLA pellets were physically mixed with TEC and fed into the microcompounder. They were compounded in molten state for 2 min, and then FB was added and melt mixed with the matrix for a further 2 min. The plastic melt was then transferred to the injection mold, and test bodies for tensile and Charpy impact testing were produced at a melt temperature of 150 °C and mold temperature of 50 °C with an injection pressure of 12 bar. For comparison, neat PLA and PLA mixed only with the plasticizer TEC were also melt compounded at 180 and 150 °C for 4 min, respectively. The formulation of composites prepared in this study is shown in Table 2.

### 2.3. Characterization

#### 2.3.1. Mechanical Properties

For the mechanical properties of neat PLA and the resultant PLA-based composites, tensile and impact strength (Charpy) properties were determined. The specimens were conditioned for 48 h in a humidity chamber with a temperature of 23 °C and humidity of 50% before the test.

The tensile tests were carried out at room temperature on dog-bone specimens in compliance with BS EN ISO 527 5A [66] by using a universal H10KT testing machine (Tinius Olsen, Ltd., Horsham, PE, USA) equipped with a 500LC laser extensometer (Tinius Olsen, Ltd., Horsham, PE, USA). The cross-head speed and gauge length were set to 5 mm/min and 20 mm, respectively. At least five specimens were tested to determine the modulus, tensile strength and elongation at break from the recorded stress vs. strain curve.

Charpy impact tests were performed on rectangular test bodies with the dimensions 80 × 10 × 4 mm, which were measured using a QC-639L impact tester (Cometech Testing Machines Co., Ltd., Taichung Hsien, Taiwan) according to the ISO 179 standard with a pendulum of 5 J. At least five specimens were tested on flatwise orientation.

#### 2.3.2. Thermogravimetric Analysis (TGA)

The thermal degradation behavior of the FB, neat PLA and PLA-based composite specimens was examined by TGA analyzer (Q500, TA Instruments, Waters LLC, Wakefield, MA, USA). The samples of 10–12 mg were placed on the platinum pans and were heated in the temperature range of 35–700 °C under a nitrogen atmosphere, using a heating rate and flow rate of 10 °C/min and 50 mL/min, respectively.

#### 2.3.3. Differential Scanning Calorimeter (DSC)

The thermal properties of neat PLA and PLA-based composite specimens were determined by DSC (Q2000, TA Instruments, Waters LLC, Wakefield, MA, USA), which was operated in a nitrogen atmosphere as the purge gas. Heating–cooling–heating cycles were performed on 5–7 mg of the specimens sealed in an aluminum pans at a rate of 10 °C/min. The first heating increased the temperature from −20 to 200 °C; then, cooling was performed from 200 to −20 °C, and the second heating as actual cycle for determination of the thermal properties of specimens was carried out from −20 to 200 °C. The crystallinity percentage (X_c_) of PLA and its blends was calculated as follows in Equation (1):

(1) $$\mathrm{Xc}=\frac{{\mathrm{DH}}_{m}-{\mathrm{DH}}_{\mathrm{cc}}}{{\mathrm{DH}}_{m}\;\left(\mathrm{PLA}\right)\times \;\mathrm{wt}\;\mathrm{PLA}}\times 100$$

where DH_m_ is the melting enthalpy, DH_cc_ is the cold crystallization enthalpy, and DH_m_ (PLA) is the melting enthalpy of 100% crystalline PLA, taken equal to 93 J/g [67]; wt PLA is the total mass fraction of PLA in the blends.

#### 2.3.4. Scanning Electron Microscope (SEM)

The distribution of the FB particles in the matrix and interfacial adhesion was examined using the SEM technique on the tensile fracture surface of the specimens. The machine was an FEI Quanta200 ESEM (FEI microscopes, Hillsboro, OR, USA) operating at an accelerating voltage of 10 kV. The specimens were gold sputtered on a sputter coater (Edwards S150B, Perth, UK) with plasma exposition of 60 s in the low vacuum mode at a pressure of 0.5 torr before the scanning to avoid electrostatic discharge during the test.

#### 2.3.5. Fourier Transform Infrared (FT-IR) Spectroscopy

The FB, neat PLA, and PLA-based composite specimens were analyzed in the frequency range of 400 to 4000 cm^−^^1^ using Nicolet iS10 FT-IR spectrophotometer (Thermo Fisher Scientific, Waltham, MA, USA). The spectra were recorded for a total of 32 scans with a resolution of 4 cm^−1^ to determine changes in chemical functionalities.

#### 2.3.6. Contact Angle Measurements

The contact angles of 5 μL water droplets on the prepared composites were measured to determine the wettability of the composite surfaces using an optical tensiometer (Attention Theta, Biolin Scientific, Gothenburg, Sweden) and the corresponding software OneAttention (Biolin Scientific, Gothenburg, Sweden). The measurements were obtained five times using a camera recorded video clip of 10 s and the final contact angle, used for analysis, was the average of five values.

### 2.4. Statistics

A one-way analysis of variance (ANOVA) at 95% confidence level was performed using SPSS 23 to evaluate the effect on mechanical properties and contact angle upon the addition of the FB and TEC in PLA. The *p*-value parameter was presented to evaluate the significance of each property.

## 3. Results and Discussion

In order to prepare specimens for the different characterization tests, PLA was replaced with FB and TEC at different ratios and molded to obtain target biocomposites. The prepared composites were then subjected to different analysis including tensile and impact test, thermogravimetric analysis (TGA), differential scanning calorimetry (DSC), Fourier transform infrared (FT-IR) spectroscopy, scanning electron microscopy (SEM), and water contact angle (WCA) test. The collected data along with the discussion on the changes experienced in the properties have been presented in the following sections.

### 3.1. Mechanical Properties

The effect of FB and TEC content on the tensile strength, elongation at break, Young’s modulus and impact strength of neat PLA and PLA-based composites is shown in Table 3.

The results indicated that the tensile strength started to decrease with increasing the amount of both FB and TEC. In fact, the tensile strength is inversely proportional to the TEC and FB loading. The neat PLA had the highest tensile strength (67.86 MPa). Plasticizing PLA with 5 to 15% TEC significantly (*p* < 0.006) decreased the tensile strength of the PLA by 23.23 to 72.92%, as shown in Table 3. The reports of Mousa et al. and Paul et al. [4,64] on PLA/date palm rachis (DPR) and PLA/microcrystalline cellulose (MCC) biocomposites, respectively, with TEC as the plasticizer showed a similar trend in tensile strength. The significant (*p* < 0.006) decrease in tensile strength (56.76 to 86.33%) with FB addition can be explained by the poor interfacial adhesion between the FB particles and the matrix that markedly develop FB agglomerates and voids during the composite fabrication, which act as stress concentration and crack initiation points in the composites. PLA samples loaded with 20% FB, as expected, are seen to have lower tensile strength, and this may be due to the presence of the higher amount of FB in the blends, which causes poorer interfacial interactions and cohesion. These interpretations are in accordance with the SEM results of the composites, which will be described in Section 3.4.

Increased elongation at break was observed by the addition of TEC in PLA and PLA/FB. This result can be related to the low molecular weight of the TEC plasticizer (276.3 g/mol), which eases the penetration of its molecules into the interface between PLA and FB, weakening the direct binding forces among the macromolecules, and in this way, the molecular chains can easily slide and move upon stress, resulting in an increase in the elongation at break [64]. The synergistic effect of TEC and FB greatly enhanced the elongation at break of the PLA/TEC/FB biocomposites. Elongation at break of composites containing FB ranged between 13 and 224% and was in all cases significantly (*p* < 0.026) higher than that of neat PLA and plasticized PLA without FB (PLA/TEC). This phenomenon can be attributed to the plasticizing effect of crude lipid (oil) content of FB. Compared with the composites containing 10% FB, a significant (*p* < 0.031) decrease in elongation at break of the composites blended with 20% FB was observed. This decrease can be explained by the higher contribution of voids in the matrix, the more concentration of stresses at FB particles and weaker interaction between the matrix and FB. The PLA-based composite with 15% TEC and 10% FB is seen to have the highest elongation at break. A similar result has been observed by Mysiukiewicz and Barczewski [68] who have reported that PLA composites filled with 10% linseed cake containing crude oil had higher elongation at break than pure PLA, while this property decreased by loading linseed cake at 20 and 30% [68].

The tensile modulus also decreased by the loading both FB and TEC, except for the PLA/5%TEC/10%FB sample. The tensile modulus of neat PLA was 4.17 GPa and showed marginal improvement for PLA mixed with 5% TEC and 10% FB (4.36 GPa); however, this was not considered as statistically significant (*p* > 0.1). What is more, the tensile modulus of composites containing 10% FB was higher than those of plasticized PLA without FB, while the lowest values were for composites mixed with 20% FB. The tensile modulus values for all samples plasticized with 15% TEC, both with and without FB, experienced a drastic drop. This finding is in agreement with Paul et al. [64], who studied the effect of three different amounts of TEC (5, 10 and 15%) on the mechanical properties of neat PLA and PLA/microcrystalline cellulose (MCC) blends.

**Figure 2:**
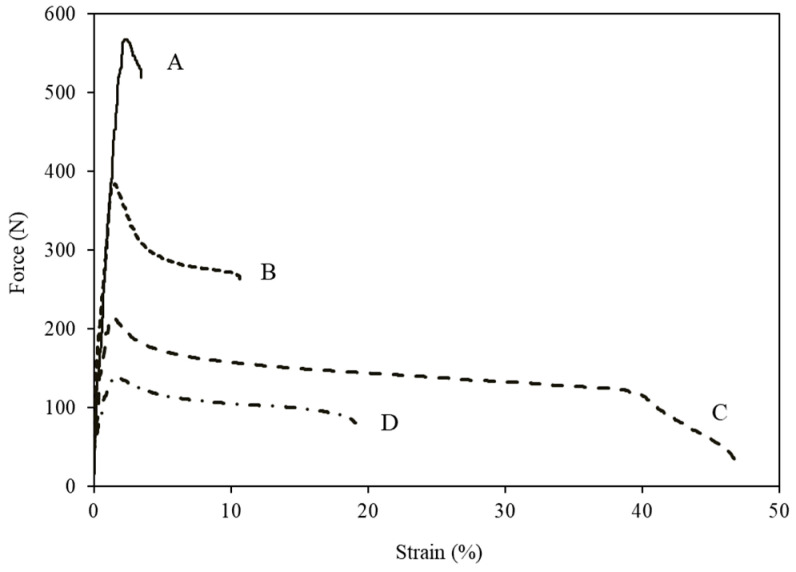
Tensile force–strain curves of (A) neat PLA, (B) PLA/10%TEC, (C) PLA/10%TEC/10%FB and (D) PLA/10%TEC/20%FB.

Force vs. strain curves of PLA specimens (Figure 2) showed that neat PLA behaves as a brittle material, whereas blending it with TEC and FB resulted in a rather ductile behavior. As can been seen, the PLA sample mixed with 10% TEC and 10% FB became more ductile than other samples. This behavior was already explained.

As shown in Table 3, the impact strength increased significantly (*p* < 0.008) with increasing the TEC content and incorporation of FB to the matrix. The impact strength of PLA-based composites blended with a plasticizer has been reported to excel that of the unplasticized biocomposites [69,70]. The plasticized PLA-based samples containing FB showed a lower impact strength in comparison with the plasticized PLA-based samples without FB. The poor interaction and compatibility between FB and matrix resulted in the creation of ubiquitous voids and gaps, which enable the process of fracture initiation and propagation; therefore, less energy is required to fracture the sample. The impact strength was lowered even more with increasing the content of FB in the composites.

### 3.2. Thermogravimetric Analysis (TGA)

**Figure 3:**
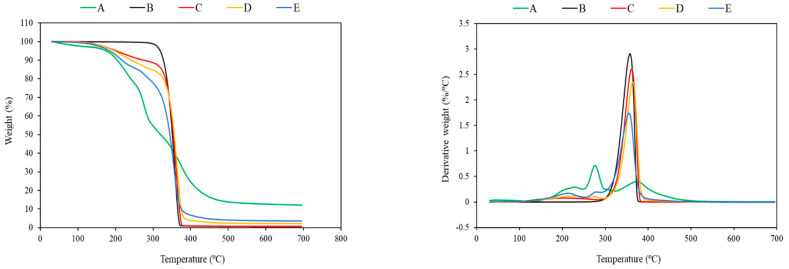
TG and DTG curves of (A) fungal biomass, (B) neat PLA, (C) PLA/10%TEC, (D) PLA/10%TEC/10%FB, and (E) PLA/10%TEC/20%FB.

The results of TGA analysis including temperature in 5% mass loss (T_5%_), temperature in 50% mass loss (T_50%_), maximum degradation temperature (T_max_) and residual mass at 700 °C are presented for all the specimens in Table 4. The thermogravimetric (TG) and derivate thermogravimetric (DTG) curves of FB, neat PLA, PLA/TEC and PLA/TEC/FB blends are shown in Figure 3.

As all the components of FB can be subjected to thermal degradation during composite processing, the processing temperature range was evaluated by TGA analysis of FB and its blends. As displayed in Figure 3, FB appears stable up to about 150 °C. For this, the processing temperature of PLA samples was lowered by TEC plasticizer up to 150 °C, and FB was not kept longer than 2 min at this temperature.

The thermal degradation of FB and composites containing FB occurred in three regions at around 200, 290, and 360–380 °C due to the presence of different components in FB as well as poor dispersion and compatibility between the FB components and the matrix. PLA, however, was degraded in a single step with a maximum degradation at 358 °C. In addition, there was a weak and broad peak at around 200–230 °C for the PLA/TEC blends, which is attributed to degradation of low-molecular-weight TEC plasticizer. The thermal degradation stages of fungal mycelium has been explained by Jones et al. [71] using a combined TGA–FTIR method. They have found that the evaporation of moisture and chemically bonded water takes place in the first region from 25 to 200 °C, the decomposition of organic compounds such as chitin, polysaccharides, proteins and lipids occurs in the second region from 200 to 375 °C, and the last region from 375 to 600 °C is related to the degradation of residual char [71]. Based on the recorded temperatures in 5% mass loss (T_5%_), the addition of TEC and FB decreased the onset degradation temperature of the PLA-based blends up to 33–143 °C, due to the earlier decomposition of TEC and FB present in the PLA matrix. However, there was no remarkable change in the T_50%_ and T_max_ of the composites on adding TEC and FB. The residual weight of FB equaled 12.12% of its initial mass after complete degradation, confirming good thermal stability. As expected, the residue of the PLA/TEC/FB samples at 700 °C was higher compared to that of the neat PLA and PLA/TEC blends due to the presence of FB. The weight loss of plasticized PLA (PLA/TEC) was also at the same level as the neat PLA.

### 3.3. Differential Scanning Calorimeter (DSC)

**Figure 4:**
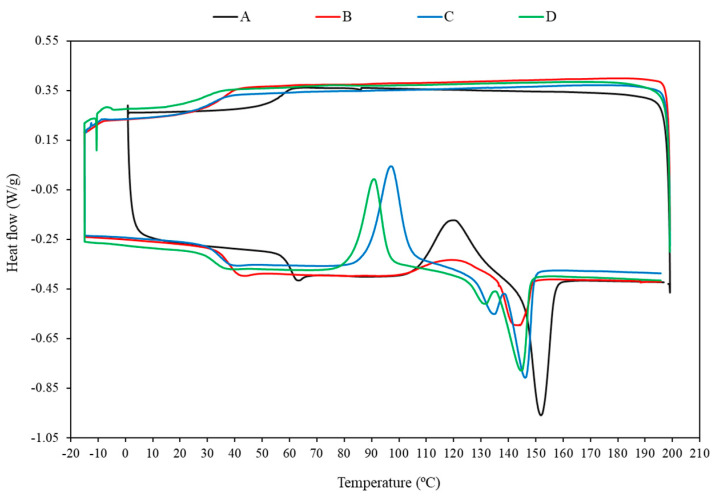
DSC curves of (A) neat PLA, (B) PLA/10%TEC, (C) PLA/10%TEC/10%FB and (D) PLA/10%TEC/20%FB.

The effects of TEC and FB addition on the thermal events of PLA samples such as the glass transition (T_g_), cold crystallization (T_cc_), melting (T_m_), cold crystallization enthalpy (DH_cc_), melting enthalpy (DH_m_) and crystallinity level (X_c_) were evaluated by DSC analysis and summarized in Table 5, whereas the DSC thermograms of neat PLA, PLA/TEC and PLA/TEC/FB blends are illustrated in Figure 4.

When PLA was blended with TEC and FB, the glass transition temperature (T_g_) was shifted toward lower values compared to the neat PLA due to the reduction in the intermolecular interactions which could cause the formation of outspread free volume at the matrix/biomass interface [11]. The glass transition temperature was found to be highest with neat PLA (55.94 °C) and lowest with PLA/15%TEC/20%FB (16.17 °C). The cold crystallization (T_cc_) and melting (T_m_) temperatures showed the same behavior as T_g_. The crystallization and melting of PLA samples blended with TEC and FB started at lower temperatures than neat PLA. The reason for the decreased the melt and glass transition temperatures and the increased degree of crystallization was ascribed to the increased chain mobility and that chain packing occurred more easily [72]. In other words, the decreased peak temperature of the cold crystallization process is in agreement with the previous studies [11,68], which have reported that linseed cake and potato pulp powder can cause a nucleating effect on the PLA and accelerate the cold crystallization process of the PLA matrix. A double endothermic peak was observed for the melting of PLA samples containing FB against the usual single peak for the neat PLA and PLA/TEC samples. Low enthalpy at crystallization and melting was obtained for the plasticized PLA samples without FB; the result implies that the TEC plasticization effect causes lower crystallization and melting temperature. The addition of the TEC and FB to the PLA matrix did not cause significant changes in the degree of crystallinity. Basically, this type of PLA is amorphous at room temperature under the experimental conditions.

### 3.4. Scanning Electron Microscope (SEM)

**Figure 5:**
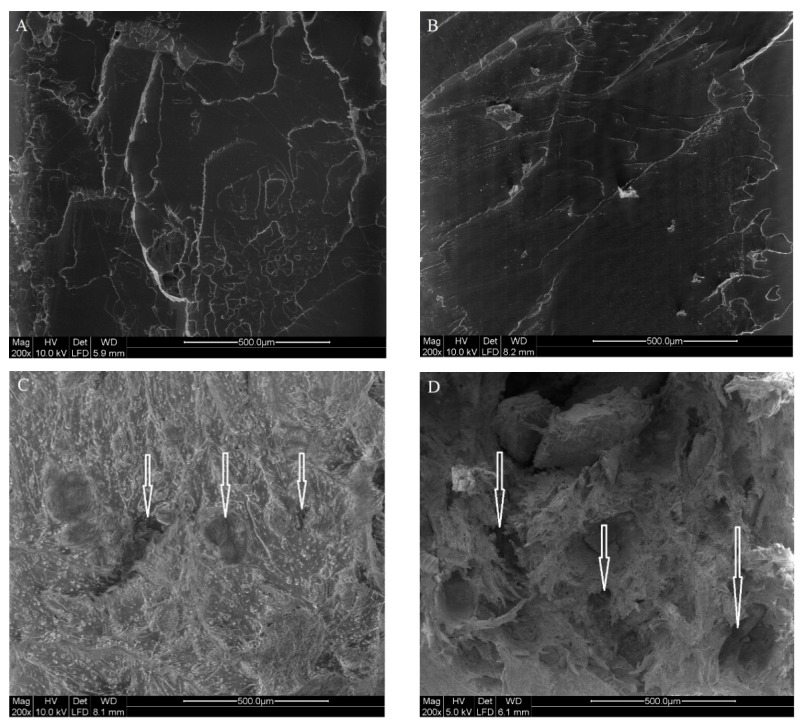
Microscopic images of (**A**) neat PLA, (**B**) plasticized PLA with 10% TEC, (**C**) PLA-based samples mixed with 10% TEC and 10% FB, and (**D**) 10% TEC and 20% FB. The arrows show gaps and voids in the biocomposites.

The morphology of the tensile fracture surface of the specimen of neat PLA, PLA/TEC and PLA/TEC/FB blends is illustrated in Figure 5.

The location of FB particles or hyphae within the matrix and the compatibility between the matrix and FB particles/hyphae can be deduced from the SEM images. PLA presented a brittle fracture pattern without significant defects and a homogeneous morphology aspect (Figure 5A,B). With the incorporation of FB, the morphology of the fracture surface changed significantly (Figure 5C,D). The addition of 10 and 20% FB to the PLA/TEC created a slightly rougher and coarser surface. FB particles can be vividly seen as irregular particles on the fractured surface. The heterogeneous dispersion of FB in the polymeric matrix resulted in the generation of aggregates as well as visible gaps and voids at the interfacial region as indicated by the white arrows. This phenomenon can demonstrate poor adhesion between the two composite compounds. As previously mentioned, FB has a complex nature with different polar and hydrophilic functional groups which can dictate low affinity with more hydrophobic PLA. The imaging findings are in agreement with the decrease in the tensile strength (Table 3). Another finding was that the particles dispersion in the blend containing 20% FB was worse, as particles were clearly pulled out of the matrix. This may justify the lower strength of the blend than that of the PLA/TEC/10%FB composite. The effect of TEC content on particles or filler dispersion in the PLA-based matrix was studied by Mousa et al. [4], Paul et al. [64] and Herrera et al. [13]. They have found that better dispersion can take place by using TEC ≥ 10 wt % in the PLA matrix.

### 3.5. Fourier Transform Infrared (FT-IR)

**Figure 6:**
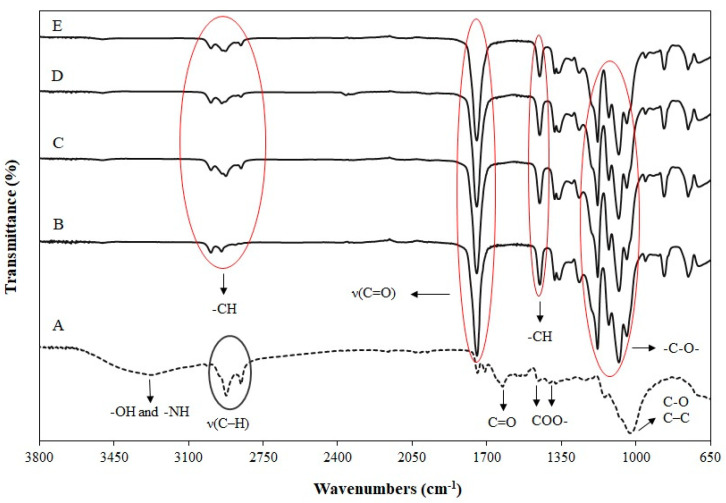
FT-IR spectra of (A) fungal biomass, (B) neat PLA, (C) PLA/10%TEC, (D) PLA/10%TEC/10%FB and (E) PLA/10%TEC/20%FB.

The effect of addition of FB in the interaction between PLA and plasticized PLA was analyzed using FTIR. Figure 6 shows infrared spectra of FB, neat PLA, plasticized PLA with 10% TEC, PLA mixed with 10% TEC and 10% FB, and PLA mixed with 10% TEC and 20% FB.

In the case of pure FB, a relatively broadened band with maximum in the region from 3262 to 3271 cm^−1^ is assigned to the -OH and -NH stretching frequencies of polysaccharides, amine, and amide present in FB. The bands in the range from 2906 to 2926 cm^−1^ and the shoulder by 2870–2884 cm^−1^ are attributed to ν(C–H) vibration aliphatic groups of fatty acids. Absorption in the range of 1641–1643 cm^−1^ corresponds to the stretching vibration of C=O groups, mainly amide (amide I). The absorption bands from 1545 to 1547 cm^−1^ are ascribed as a deformation -NH vibration of amides (amid II). The C=O symmetric stretching of COO- groups appeared in the range of 1413–1415 and 1374 cm^−1^. FB can have intensive absorption band characteristics for C-O and C–C vibration (1200–950 cm^−1^), referring to the presence of polysaccharides [73]. In general, all PLA samples exhibited a characteristic peak around 1750 cm^−1^, corresponding to the carbonyl stretching vibration ν(C=O) band of PLA. The bands between 2990 and 2850 cm^−1^ belong to the -CH- asymmetric and symmetric stretching vibrations of CH_3_ groups in the side chains, whereas their bending vibration was observed at 1454 cm^−1^. The –C–O– bond stretching in –CH–O– and in –O–C=O of PLA were detected at 1180 and 1080 cm^−1^, respectively. No new peaks were observed when TEC was added to PLA or when FB was added to the PLA/10%TEC blend, confirming no chemical interaction between TEC and PLA or FB and PLA/TEC [13,15].

### 3.6. Contact Angle Analysis

The hydrophilic or hydrophobic nature of the surface of the PLA-based blends was examined by contact angle analysis, and the results are given in Table 6. In this regard, the neat PLA and its blends showed a hydrophilic surface, as the contact angle was less than 90°. No significant change was observed when PLA was plasticized with 10% TEC. A similar finding was obtained by Paul et al. [64], who have reported that the addition of 10 wt % TEC in PLA leaves the water contact angle (WCA) practically unchanged (WCA 99.5 ± 1.6° for pure PLA films and 101.3 ± 2.0° for PLA/10% TEC). It was assumed that the wettability of PLA-based samples improves by FB addition. However, there was no significant difference (*p* > 0.1) between the contact angle of the neat PLA, plasticized PLA with 10% TEC, and PLA mixed with 10% TEC and 10% FB, while the addition of 20% FB to the plasticized PLA resulted in a significant decrease in the contact angle (*p* < 0.02) as the surface tendency to form hydrogen bonding with water molecules increased due to the presence of more polar/functional groups. It is clear that the blending of PLA with natural polymers, especially hydrophilic polymers such as protein, starch, cellulose, etc., improves the water absorbance properties of the PLA-based blends by reducing the ratio of hydrophobic moieties. On the other hand, the accumulation and penetration of water in the voids and microcracks within the PT10F20 sample can be another reason for the decrease in the sample’s contact angle.

## 4. Conclusions

In this study, FB and TEC were successfully blended with PLA using extrusion followed by injection molding to prepare green composites. The present results clearly show that the addition of TEC and FB had a great influence on the thermal and mechanical properties. The obtained biocomposites showed the decrement of glass transition, crystallization and melting temperature compared to that of the neat PLA. It was found that there was no reinforcing effect on the PLA matrix, but the incorporation of FB and TEC to PLA makes the blend more ductile. The PLA-based composites with 10% TEC and 10% FB showed a greater increase in flexibility and ductility and enhanced impact strength. The blend did not also cause significant changes in the contact angle. It is worth mentioning that the composition of FB is expected to depend on the growth media, the environmental conditions, as well as the fungus type and age. For this reason, the present study provides only an overall description of the properties of PLA-based biocomposites, which could slightly change with varying the original materials. Future investigation of the effect of addition of coupling agents and/or surface modification of FB via grafting on the adhesion and compatibility between the PLA matrix and FB is proposed to further optimize the strength properties of the final biocomposite.

## Figures and Tables

**Table 1:** The composition of fungal biomass (FB).

Composition	Value (%)
Moisture	3.5 ± 0.18
Ash	3.8 ± 0.13
Total lipid	21.3 ± 0.61
Crude protein	37.6 ± 1.20
Other components ^1^	33.8 ± 1.53

^1^ Composed mainly of polysaccharides such as cellulose, chitin, and α- and β-glucans.

**Table 2:** Formulations of the fabricated composites in this study.

Sample Code	Poly(lactic acid) (PLA) %(*w*/*w*)	Triethyl Citrate (TEC) %(*w*/*w*)	Fungal Biomass (FB) %(*w*/*w*)
Neat PLA	100	-	-
PT5	95	5	-
PT10	90	10	-
PT15	85	15	-
PT5F10	85	5	10
PT10F10	80	10	10
PT15F10	75	15	10
PT5F20	75	5	20
PT10F20	70	10	20
PT15F20	65	15	20

**Table 3:** Mechanical properties of neat PLA and PLA-based composites.

Sample Code	Tensile Strength (MPa)	Elongation at Break (%)	Young’s Modulus (GPa)	Charpy Impact Strength (kJ/m^2^)
Neat PLA	67.8 ± 1.0	3.8 ± 0.6	4.17 ± 0.60	15.31 ± 1.06
PT5	52.1 ± 1.0	6.3 ± 0.9	3.50 ± 0.94	19.44 ± 3.05
PT10	43.2 ± 1.7	10.2 ± 2.0	2.33 ± 0.78	24.52 ± 2.96
PT15	18.4 ± 2.9	199.8 ± 11.7	0.43 ± 0.06	92.67 ± 3.60
PT5F10	29.3 ± 1.5	13.0 ± 1.3	4.36 ± 0.85	10.85 ± 1.42
PT10F10	24.1 ± 1.0	42.9 ± 7.0	2.85 ± 0.61	12.72 ± 0.79
PT15F10	10.9 ± 1.1	224.0 ± 14.5	0.44 ± 0.06	65.92 ± 4.36
PT5F20	25.5 ± 3.7	10.8 ± 1.6	3.19 ± 0.61	8.36 ± 1.56
PT10F20	19.7 ± 4.0	25.1 ± 4.3	2.24 ± 0.49	12.12 ± 0.75
PT15F20	9.3 ± 1.7	200.5 ± 10.5	0.07 ± 0.02	41.21 ± 4.75

**Table 4:** TGA analysis parameters of neat PLA and PLA-based composites.

Sample Code	T_5%_ (°C)	T_max_ (°C)	T_50%_ (°C)	Residue (%)
Fungal biomass	175.7	291.6	320.1	12.1
Neat PLA	318.9	358.3	351.1	0.7
PT5	285.6	368.9	362.4	0.8
PT10	202.9	362.6	354.5	0.7
PT15	196.0	349.2	340.8	0.7
PT5F10	222.9	363.0	357.5	2.1
PT10F10	196.8	361.5	355.5	2.2
PT15F10	178.9	361.4	354.0	2.1
PT5F20	212.5	361.4	355.0	3.5
PT10F20	186.8	360.2	350.5	3.3
PT15F20	175.8	361.0	350.2	3.2

**Table 5:** Transition temperatures and enthalpies of neat PLA and PLA-based blends.

Sample Code	T_g_ (°C)	T_cc_ (°C)	T_m_ (°C)	ΔH_cc_ (J/g)	ΔH_m_ (J/g)	X_c_ (%)
Neat PLA	55.9	120.3	151.9	24.5	25.3	0.9
PT5	43.5	124.7	148.5	5.2	5.4	0.2
PT10	30.5	119.4	144.1	9.3	9.4	0.1
PT15	21.4	110.4	137.9	14.7	15.1	0.5
PT5F10	36.7	106.9	149.0	30.9	31.2	0.4
PT10F10	27.8	96.8	145.8	26.9	27.6	1.0
PT15F10	20.9	85.6	141.9	21.8	22.0	0.2
PT5F20	34.5	99.9	147.6	27.1	28.4	1.8
PT10F20	25.5	90.3	144.5	24.0	24.2	0.3
PT15F20	16.8	82.9	142.5	20.2	20.5	0.4

**Table 6:** Water contact angle (WCA) of PLA-based samples.

Sample Code	WCA Mean [°] 0 SEC	WCA Mean [°] 10 SEC
Neat PLA	73.68 ± 2.81	73.19 ± 2.54
PT10	73.75 ± 2.64	72.18 ± 2.52
PT10F10	72.76 ± 3.85	72.46 ± 3.97
PT10F20	64.35 ± 3.47	60.63 ± 4.65

## Data Availability

Not applicable.
